# Intelligence and Creativity: Mapping Constructs on the Space-Time Continuum

**DOI:** 10.3390/jintelligence9010001

**Published:** 2020-12-30

**Authors:** Giovanni Emanuele Corazza, Todd Lubart

**Affiliations:** 1 DEI-Marconi Institute for Creativity, University of Bologna, Viale Risorgimento 2, 40136 Bologna, Italy; 2 LaPEA, Université de Paris and Univ Gustave Eiffel, F-92100 Boulogne-Billancourt, France; todd.lubart@u-paris.fr

**Keywords:** intelligence, creativity, definition of intelligence, definition of creativity, tightness, looseness, conceptual space, measurement of intelligence, measurement of creativity, relationship between intelligence and creativity

## Abstract

This theoretical article proposes a unified framework of analysis for the constructs of intelligence and creativity. General definitions for intelligence and creativity are provided, allowing fair comparisons between the two context-embedded constructs. A novel taxonomy is introduced to classify the contexts in which intelligent and/or creative behavior can be embedded, in terms of the tightness vs. looseness of the relevant conceptual space S and available time T. These two dimensions are used to form what is identified as the space-time continuum, containing four quadrants: tight space and tight time, loose space and tight time, tight space and loose time, loose space and loose time. The intelligence and creativity constructs can be mapped onto the four quadrants and found to overlap more or less, depending on the context characteristics. Measurement methodologies adapted to the four different quadrants are discussed. The article concludes with a discussion about future research directions based on the proposed theoretical framework, in terms of theories and hypotheses on intelligence and creativity, of eminent personalities and personality traits, as well as its consequences for developmental, educational, and professional environments.

## 1. Introduction: The Complex Relationship between Intelligence and Creativity

Intelligence and creativity can arguably be considered to be the most important, and perhaps most distinctive, constructs that characterize human existence in terms of knowledge acquisition, knowledge production, and knowledge-based behavior. In other words, intelligence and creativity are two fundamental epistemological constructs, largely determining the cultural richness of our species. Unsurprisingly, they have both been the subject of extensive study and research, not exclusively but primarily in the fields of psychology, philosophy, sociology, anthropology, and economics. In this article we will focus on psychological science, but the theoretical framework that we will introduce is not devoid of implications on all related disciplines of human and social sciences.

It should be readily agreed that intelligence and creativity are not orthogonal constructs. For example, introducing novel paradigms in science, such as the theory of relativity by Einstein, requires exceptional levels in both constructs, and it may be hard to determine whether one prevails over the other. On the other hand, given the importance of intelligence and creativity in all human endeavors, and specifically in educational and professional environments, it would be very important to have a clear understanding of the extant relationships between these two fundamental constructs.

Notably, this is still an open problem today, as testified by the abundant literature on the subject (Benedek et al. 2018; Getzels and Jackson 1962; Guilford 1956, 1967, 1988; Jung et al. 2009; Karwowski et al. 2016; Karwowski and Gralewski 2013; Kaufman and Plucker 2011; Lubart 2003; Nusbaum and Silvia 2011; Plucker et al. 2015; Plucker et al. 2017; Silvia 2015; Sligh et al. 2005; Sternberg 1999; Sternberg and O’Hara 1999; Wallach and Kogan 1965). A few quotations from this literature are in order to understand the level of this challenge. The importance of the issue is underlined by Sternberg (1999, p. 87): “Despite a substantial body of research, psychologists still have not reached a consensus on the nature of the relation between creativity and intelligence […]. All possible set relations between creativity and intelligence have been proposed, and there is at least some evidence to support each of them. […] The question is theoretically important, and its answer probably affects the lives of countless children and adults. We therefore need elucidation of good answers as soon as possible.” This opinion is confirmed by Kaufman and Plucker (2011, p. 779): “Researchers and theorists do not believe that intelligence and creativity are completely orthogonal, but beyond that, the exact nature of that relationship remains an open question.”

As Silvia (2015) has put into evidence, there have been significant shifts of opinion in the last decades, ranging from statements of negligible to very low correlation between the two constructs (Getzels and Jackson 1962; Wallach and Kogan 1965), to more modern assessments of substantial correlation, based on improved measurement with latent variable models, more correct scoring of originality, and revelations from cognitive neuroscience (Nusbaum and Silvia 2011). It should also be underlined that correlation in itself is not the sole, and perhaps not the most adequate, measure of interconnection between these two constructs. This is because their relation may well be non-linear, as exemplified by the Threshold Hypothesis (TH) or the Necessary Condition Hypothesis (NCH). The TH states that up to an IQ threshold, a positive correlation between intelligence and creativity should be found, whereas above that threshold this should disappear, or at least significantly reduce its value (Karwowski and Gralewski 2013; Jauk et al. 2013; Jung et al. 2009; Sligh et al. 2005). On the other hand, the NCH states that intelligence should be a necessary (but not sufficient) condition for creativity, which appears to be confirmed by data even more robustly than the TH (Karwowski et al. 2016). In their criticism of the TH, Karwowski et al. (2016) underline the fact that the hypothesized threshold appears to vary considerably depending on the methodology selected for the analysis. At any rate, the fact that much work is still in front of us is confirmed by the neuroscience community, as stated by Benedek, Jung, and Vartanian when presenting a Special Issue of Neuropsychologia devoted to the neural bases of creativity and intelligence (Benedek et al. 2018, p. 3): “The contributions to this Special Issue reveal evidence for both common and unique brain processes in creativity and intelligence. […] they also raise several important questions that will stimulate future research in this dynamic field.”

Whereas we must recognize the great value of the existing contributions to this topic, we believe that an important reason why difficulty still remains in establishing the relationship between intelligence and creativity is the apparent lack of a theoretical framework that: (i) is built on compatible and comparable definitions for the two constructs; (ii) affords fair observations under characteristic contextual conditions of human behavior.

The first point, concerning the definitions of intelligence and creativity, is critical as it bears directly upon the results of any comparison. Leaving definitions implicit and only describing operationalization through measurements is not sufficient, because the understanding that can be derived may change simply by changing the selected measures. On the other hand, the importance of controlling for context has been clearly emphasized by Plucker, Lim, and Lee in their work on implicit theories (Plucker et al. 2017, p. 398): “The relationship between creativity and intelligence, at least as represented in people’s implicit theories of these constructs, may behave differently based on the context in which the theories are applied and on the construct that is predominant in the individual’s mind when they apply their theory.” More generally, it should be noted that the consideration of context is one of the main strategies for theory development, as indicated by Wicker (1985) to overcome apparently unsolvable struggles.

The aim of our work is thus to address the points (i) and (ii) outlined above by introducing a theoretical framework based on compatible and comparable definitions for the intelligence and creativity constructs, as well as on a contextual domain for their existence identified as the *space-time continuum*. The latter represents in a novel way both the structure of the engaged conceptual space S, in which solutions/responses are sought, and the temporal domain T, in which the intelligence and/or creativity process occur. The axes of this space-time continuum, and therefore the characteristics of S and T, are described through a taxonomy based on the concepts of *tightness-looseness*, which have been inspired by the work of Gelfand et al. (2011) about attributes of societies. Here we extended the application of these attributes to the conceptual space and the available time span for the human episode under observation. As shown later, both space S and time T can vary in continuity from extreme tightness to extreme looseness. By crossing these two dimensions, the space-time continuum is shown to contain four quadrants: tight space-tight time, loose space-tight time, tight space-loose time, and loose space-loose time. These quadrants allow to map and control the context in which intelligence and creativity constructs are activated and compared in thought experiments, in laboratory experiments, in controlled environments, or in the field.

In the aforementioned quote of Sternberg’s work (Sternberg 1999, p. 87), it is stated that all possible set relations between intelligence and creativity can be hypothesized and justified by measurements. The theoretical approach we propose can serve to unravel this intricate skein: in fact, the space-time continuum allows to distinguish domains of human activity where intelligence is definitely useful whereas creativity might be detrimental (i.e., the tight space-tight time quadrant), areas where creativity may flourish whereas intelligence might drive one away from the effort (i.e., the loose space-loose time quadrant), as well as domains in which the two constructs cooperate and are largely overlapping in meaning and functionality (i.e., the hybrid quadrants with mixed looseness and tightness). A further important contribution that can be found in this article consists of showing that for each of the four quadrants in the space-time continuum there exist different measurement and testing techniques. This allows to map extant scientific approaches to provide further clarification about what otherwise could be classified as mixed results. The many possible avenues that can be further explored basing one’s scientific enquiry on our proposed theoretical framework are addressed in the Discussion and Conclusions to the paper.

## 2. Definitions of Intelligence and Creativity

It is our firm belief that a theoretical framework allowing a proper, scientifically-based comparison between intelligence and creativity must start from giving definitions for the two constructs. The motivation behind this statement is clear: the result of the comparison will depend fundamentally on the adopted definitions, which therefore must be considered to be the basis of any theoretical framework.

On the other hand, in the vast extant literature concerning the relationship between intelligence and creativity, a contribution stating explicit definitions for the two constructs is hard to find. It can certainly be argued that even if an experimental study does not mention explicitly the definitions of the two constructs, the empirical approach which is undertaken provides them implicitly. This is an operational-oriented approach, which may be acceptable when a study is dedicated to a single construct, but even in this case it is not devoid of misinterpretation in terms of excessive extrapolation or generalization. To describe the associated danger, consider for example the fact that many articles that adopt the Alternative Uses Test (AUT) (Christensen et al. 1960) as a measure of divergent thinking make claims and draw conclusions about the *creativity* of the participants: this extrapolation is excessive and therefore logically incorrect, as it is well known that divergent thinking is only one (albeit a very important one) of the cognitive components that might be used in certain phases of the creative thinking process. Indeed, the creative process includes many other aspects that are not measured by the AUT (Runco 2008). It is possible that a person who has excellent AUT performance still shows poor creativity, for example because he or she is unable to formulate problems, ask the right questions, extract value from original ideas, bring them into reality, etcetera.

Given this problematic situation for the study of a single construct, it should then be apparent that working with implicit definitions is not only subject to criticism, but rather ill-posed and possibly misleading when the objective is to compare two different constructs. In this regard, it is relevant to report on the conclusions of Plucker et al. (2015, ch. 19, p. 289): “Definitions are critically important when dealing with psychological constructs, as the way in which each construct is conceptualized and assessed will have a significant impact on any empirical results when comparing two or more constructs. […] the range of creativity and intelligence definitions makes the complexity of possible intelligence-creativity relationships unsurprising. Few people believe creativity and intelligence are completely unrelated, but the nature of any relationship—even with so much research already conducted—is an open question.” However, even in Plucker et al. (2015) the definitions for intelligence and creativity are not actually given; rather, the framework of analysis proposed by Sternberg and O’Hara (1999) is adopted, foreseeing the following five set relations: creativity as a subset of intelligence, intelligence as a subset of creativity, creativity and intelligence as overlapping sets, creativity and intelligence as coincident sets, and creativity and intelligence as disjoint sets. However, these possible set relations cannot be considered as definitions for the constructs. Not surprisingly, it has been shown (Plucker et al. 2015; Sternberg and O’Hara 1999) that empirical evidence can be found to justify each of these five set relations. In essence, with this approach we are left off where we started in studying the relationship between intelligence and creativity.

The respective pools of extant literature on the respective definitions of intelligence and creativity are both vast and characterized by significant variability. For intelligence, see (Colvin et al. 1921; Detterman and Sternberg 1986; Legg and Hutter 2007) and the references therein; for creativity, see (Corazza 2016; Mayer 1999; Parkhurst 1999; Rhodes 1961; Runco and Jaeger 2012; Sternberg 1988) and the references therein.

The first element to note is that both definitions may have different levels: they can focus on cognitive components, individual behavior, sociocultural aspects and contexts, or a mixture of these elements. In selecting our definitions for the purpose of establishing a mutual relationship, it is therefore essential that both constructs be defined *at the same level*. Failing to meet this basic requirement would turn immediately even the best and most accurate scientific work into an apples and pears comparison. Symmetry is essential in order to draw any fair conclusion in comparing the two constructs. For example, it would be asymmetric to define intelligence through its necessary cognitive components and creativity through the socioculturally acclaimed achievements in one’s career. The chosen level of definition is critical as well; as a general indication, the higher the level of the definition the wider the coverage of the overall phenomenon, but also the higher the difficulty in the operationalization of the definition. As a consequence, a good criterion may be to select a definition level as high as possible, given that its operationalization is feasible and effective.

**Figure 1:**
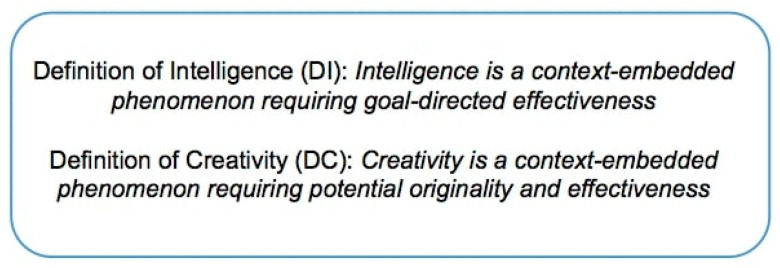
Definitions of Intelligence (DI) and Creativity (DC) adopted in the proposed theoretical framework.

The selected definitions we present in the following have to be considered as the fundamental axioms of the theoretical framework that will be built upon them, and all the discussion and the significance of the conclusions that will be drawn should be interpreted by the reader in light of these definitions. This is unavoidable, as should be very clear given the above discussion. At the same time, even if the reader would give a different definition for either or both of the two constructs, or no definition at all, we invite to use the selected definitions as working assumptions, and see what the consequences are in terms of the resulting theoretical framework. The proposed definition of intelligence (DI) and definition of creativity (DC) are provided in Figure 1. We begin by commenting each definition on its own, and then we address them jointly.

### 2.1. Definition of Intelligence DI

Considering the intelligence of an individual actor, the proposed definition DI places its emphasis upon three fundamental elements: (i) the goals, established by the actor himself/herself or by persons interacting with the actor; (ii) the context in which these goals are set, in view of their adaptiveness; (iii) the effectiveness of the actor’s behavior in pursuing these goals. In terms of its conceptual level, definition DI focuses on the actor’s behavior as embedded in a sociocultural context. By properly interpreting goals, context, and measures of effectiveness, definition DI can be shown to contain most of the existing definitions for intelligence.

Starting from seminal classic contributions, several definitions of intelligence were reported as outcomes of the 1921 symposium organized by the editors of the Journal of Educational Psychology (Sternberg 1990, p. 35), as well as of the 1986 symposium organized by Detterman and Sternberg (Sternberg 1990, p. 36). Let’s consider a few specific examples. In 1921, Thorndike defined intelligence as “*the power of good responses from the point of view of truths or facts*.” In terms of definition DI, the goal is to provide responses that can be judged to be “good”, in the context of a search for “truths or facts”, which are sociocultural categories, and the effectiveness is quantified as the “power” to provide the wanted good responses. Additionally, in 1921 Colvin defined intelligence as “*having learned or ability to learn to adjust oneself to the environment*”. In terms of definition DI, the “goal” and “context” are here specified as “adjusting to the environment”, and the “effectiveness” is having learned and the ability to continue to learn dynamically how to adjust to variable contexts. Still in 1921, Thurstone proposed a quite long definition for intelligence as *“the capacity to inhibit an instinctive adjustment, the capacity to redefine the inhibited instinctive adjustment in the light of imaginally experienced trial and error, and the capacity to realize the modified instinctive adjustment in overt behavior to the advantage of the individual as a social animal.”* In terms of definition DI, the “goal” is “rational adjustment”, the “context” is the implicit social environment to which the “individual as a social animal” must adjust, and the “effectiveness” is related to three elements: the capacity to inhibit the instinct that might lead to non-rational action, the ability to imagine and perform abstract trial-and-error of alternatives, the capacity to transform abstract thought into action.

In the 1986 event, Baron defined intelligence as *“the set of abilities involved in the achievement of rationally chosen goals”* (Sternberg 1990, p. 43). The correspondence with definition DI comes through the following qualifications: the rationality of the chosen goals must be judged against the context embedding the phenomenon; whereas Baron’s definition focuses on a set of abilities, definition DI points at the actual exploitation of these abilities for the effective pursuit of the rationally chosen goals in the context. In 1986, Carroll argued (Sternberg 1990, p. 44) that the domains to which intelligence is applied are threefold: academic and technical, practical, and social, and that the exact nature of intelligence may depend upon society. This argument by Carroll is clearly in line with definition DI in conceiving intelligence as a context-embedded phenomenon.

Still at the 1986 symposium, Gardner proposed his view of multiple, independent intelligences. In view of definition DI, each of the domains identified by Gardner (linguistic, logical-mathematical, musical, spatial, kinesthetic, intrapersonal, interpersonal) is a distinct epistemological and behavioral “context”, characterized by diversified “goals”, requiring relevant abilities for their effective pursuit. However, it should be noted that definition DI does not imply in any way that these multiple sets of abilities are “independent”, which is perhaps the main criticism that has been raised about the theory of multiple intelligences. To conclude our brief review of the 1986 symposium, Sternberg defined intelligence as *“providing a means to govern ourselves so that our thoughts and actions are organized, coherent, and responsive to both our internally driven needs and to the needs of the environment”* (Sternberg 1990, p. 49). This is perfectly compatible with definition DI, provided that the “goals” are interpreted as needs, the nature of which is internal as well as context-driven, and the effectiveness is related to self-governance of both thought and action.

Let’s consider two more recent contributions on the topic. Gottfredson (1997), in a contribution with 52 signatories, proposed the following long definition: “*Intelligence is a very general mental capability that, among other things, involves the ability to reason, plan, solve problems, think abstractly, comprehend complex ideas, learn quickly and learn from experience. It is not merely book learning, a narrow academic skill, or test-taking smarts. Rather, it reflects a broader and deeper capability for comprehending our surroundings—‘catching on,’ ‘making sense’ of things, or ‘figuring out what to do‘*”. This definition is fully compatible with definition DI: comprehending the surroundings to make sense of things and figure out what to do is tantamount to setting goals in a context-embedded phenomenon, and the effective pursuit of these goals will require reasoning, planning, problem solving, etcetera. Finally, Legg and Hutter (2007) collected around 70 different definitions for intelligence, both in the realm of psychology and in that of artificial intelligence. It is not possible here to consider all of them and show how definition DI is general enough to cover the different nuances of each definition. We limit ourselves to a single example, coming from psychologist Anastasi (Legg and Hutter 2007, p. 4; see also Anastasi 1992): “*Intelligence is not a single, unitary ability, but rather a composite of several functions. The term denotes that combination of abilities required for survival and advancement within a particular culture*”. In terms of definition DI, we recognize as the context-embedded goal that of surviving and advancing within a particular culture, and the effectiveness of the pursuit of this goal as dependent on a combination of abilities.

As a conclusion of this analysis, we consider that definition of intelligence DI is sufficiently comprehensive and appropriate as an axiom upon which to build a theoretical framework for the comparison of the intelligence and creativity constructs.

### 2.2. Definition of Creativity DC

Considering the definition of creativity, the literature on the subject is as abundant as that for the definition of intelligence, with both classic and modern contributions (see for example Corazza 2016; Kharkhurin 2014; Mayer 1999; Parkhurst 1999; Rhodes 1961; Stein 1953; Sternberg 1988; Weisberg 2015, and references therein).

A so-called standard definition (SD) of creativity does exist (Runco and Jaeger 2012, p. 92): *“Creativity requires both originality and effectiveness”*. This definition is in line with most of the definitions that can be found in the relevant literature, although a debate is still active on the number of criteria required to identify creativity (e.g., Kharkhurin 2014; Weisberg 2015). As can be seen, the terminology is not far from the definition DC we proposed above, but there are fundamental differences. In fact, as argued by Corazza (2016), rather than the complete phenomenon of creativity, SD defines only a form of *creative achievement*, i.e., an instance in which there is a positive recognition by some observer of the originality and effectiveness of a course of thoughts and/or actions. This is similar to taking a picture of a wave at its highest peak: it might be the most important instant, but it does not represent the entire undulatory phenomenon, missing the dynamics that have led to that creative achievement.

For this reason, it can be argued that if one adopts definition SD for creativity, the ensuing theoretical framework is static, i.e., based on stable recognition. On the other hand, creativity is fundamentally a dynamic phenomenon, and there are at least two key reasons to modify the standard definition of creativity to account for this dynamism. The first reason is that a creative process is an activity characterized by risk, in the sense that the creative outcomes cannot be foreseen a-priori. If they were, they could not be original, and hence creativity would be denied. This means that when an actor is engaged in a creative activity, perhaps challenging very consolidated state-of-the-art knowledge in a domain (an act which might not be considered “intelligent”), it cannot be guaranteed that originality and effectiveness will emerge.

As in (Corazza 2016), we identify the event in which the goals of the creative process are not (yet) achieved as a state of *creative inconclusiveness*. The latter should not be intended as failure: creative inconclusiveness is an integral part of the process, and indeed all great creative geniuses share the characteristics of having been able to persist and emerge out of some form of inconclusive conditions, that perhaps others considered insurmountable and sufficient to abandon. Creative inconclusiveness is an extremely important state in the process, hence the theoretical framework and the definition of creativity upon which the theoretical framework is built must be able to contain it.

The second reason why a static framework is insufficient is that even when outcomes are positively produced from a creative process, the estimation of their value in terms of originality and effectiveness is potentially an indefinite, never ending process. Any assessment, for example in terms of a score in a test, or the appreciation by critics of a work of art, should be considered as a static picture of a dynamic scene: useful, important, but limited to a specific time, space, and culture. In the history of the arts, science, and technology, there are innumerable examples of tangible and intangible ideas and works that were harshly criticized or dismissed at first, only to become seminal to creative disruptions several years later, sometimes tens or hundreds of years later.

Two examples are in order: paintings by Vincent Van Gogh were completely neglected by critics and the public during the unfortunately short course of the painter’s life. Indeed, Vincent was able to sell only one of his paintings during his life. Success would only come posthumously thanks to the efforts of Johanna Bonger, the wife of his brother Theo, who organized the first Van Gogh expositions and had to fight against the critics of the time. Our second example refers to the person who was arguably the most creative in the history of Homo Sapiens: Leonardo da Vinci, who produced creative work in more than twenty different disciplines across the arts, science and technology. The fact is that most of Leonardo’s ideas remained inconclusive in the course of his lifetime, only to be recognized as anticipatory of modern ideas (such as for example the helicopter, the tank, optical lenses, …) hundreds of years later. In essence, the value of creative ideas is a dynamic entity, and the definition of creativity must be able to subsume this fact.

For these two fundamental reasons, Corazza (2016) introduced the dynamic definition of creativity, which foresees that “*creativity requires potential originality and effectiveness*”. The difference comes from the introduction of “potential” inside the definition of creativity. The level of potential depends on the challenge that is posed to extant knowledge in the course of the creative process. If one remains within the comfortable boundaries of extant knowledge, focusing on brilliant and correct answers, the potential tends to zero. Potential for originality and effectiveness requires excess energy to go beyond previous knowledge.

When this potential is realized and recognized, we have an instance of creative achievement, as in the definition of creativity SD, which is therefore subsumed by the dynamic definition of creativity. Hence, all other definitions for creativity which are compatible with definition SD are subsumed by the dynamic definition of creativity. On the other hand, when the potential remains latent, is not recognized either by the actor or by the outside world, or the true value of the outcomes does not emerge or fluctuates to a minimum, the process remains or enters in a state of creative inconclusiveness, which could in reality be a very important part of the phenomenon. This part of the process escapes definition SD but is properly included in the dynamic definition of creativity. Potential is there, but it remains latent.

The definition of creativity DC introduced in this article, in agreement with (Corazza and Lubart 2020), contains the dynamic definition of creativity and adds explicitly that creativity is a context-embedded phenomenon, to place the emphasis on the fact that our understanding of the construct is tightly related to the cultural and social environment, and to ensure that the level of the definition DC is identical to that of definition DI. The importance of this addition is therefore apparent when we consider the two definitions together, as discussed in the following section.

### 2.3. Comparisons of Definitions DI and DC

Considering now the definitions for the two constructs contained in Figure 1 simultaneously, we start to note that they are built with two explicit similarities:

- both phenomena are “context-embedded”;
- both definitions include the “effectiveness” keyword, although with fundamental differences, requiring detailed discussion.

First, as we will show in the following the effect of embedding a behavior in a specified context produces different, sometimes opposite, effects in terms of our understanding of that behavior as intelligent or creative. In other words, whereas both constructs are context-embedded, their mutual relationship is strongly dependent on the characteristics of the embedding context, and our possibility to discriminate clearly between intelligence and creativity depends critically on our ability to find those contexts in which the two constructs do not overlap. This may arguably be one of the main properties of the proposed theoretical framework.

Second, considering the “effectiveness” keyword, in terms of intelligence this should be interpreted as the successful attainment of the domain-specific goals that have been set out a-priori; on the other hand, in terms of creativity this points to a search for an entity that a-posteriori might potentially show appropriate value in terms of a domain-specific functionality and/or performance, to be compared to extant state-of-the-art entities in that domain. In other words, if in a creative process an unexpected outcome is generated which shows some effectiveness with respect to a problem, but a state-of-the-art solution exists with far superior effectiveness, the ideated outcome would still be considered inconclusive (but fully part of the dynamics of a creative process).

This is only an example of the dynamics that allow us to discriminate between the two constructs, and these would not be observable if one adopted the SD definition for creativity. The dynamic definition of creativity is thus essential in bringing up the a-priori vs. a-posteriori variability that turns out to be key in contrasting the intelligence and creativity constructs.

In terms of explicit differences between the two construct definitions provided in Figure 1, the following two elements stand out:

- Intelligence requires goal-direction, whereas creativity does not *necessarily* require goal-direction.
- Creativity requires potential originality, whereas intelligence does not *necessarily* require potential originality.

Now, it is important to underline that, even though it is not necessarily required, in certain contexts creativity can also be goal-directed, as for example when solving a wicked problem. Indeed, creative problem-solving is a goal-directed exercise which requires a creative process, and effectiveness in finding a solution is a very important feature. In these contexts, creativity and intelligence become hardly distinguishable; in fact, many definitions of intelligence include complex problem solving as a main element (e.g., see the aforementioned definition by Gottfredson 1997). However, the definition DC allows also to consider those situations of goal-free exploration, unexpected turns of events, and serendipitous findings which are not goal-directed by definition, or at least for which goals are fuzzy, a-priori.

In a similar fashion, even though intelligent behavior does not require originality as a necessity, in particularly challenging contexts the use of intelligence may lead to very original solutions. Also in these contexts, the two constructs of intelligence and creativity become nearly undistinguishable. But definition DI encompasses also that majority of contexts in which goals, behavior and responses sit all within the boundaries of extant knowledge; intelligence is conducive to brilliant and fast responses whose potential for originality tends to be null, and rightly so. In these contexts, creativity is very distinct from intelligence and it may actually introduce noise, e.g., in the form of unnecessary mind wandering.

One final consideration on energy: both constructs involve the expenditure of significant levels of energy, but while according to definition DI intelligence is driven by the objective to spend the minimum energy necessary to reach established goals effectively, for creativity the quest for potential originality contained in definition DC might lead to very significant excess energy expenditures. Therefore, involved energy is another key in distinguishing the two constructs.

This initial discussion, entirely based on the definitions for the constructs, is already rich in indications about how to clarify the relationship between intelligence and creativity. However, going further in depth requires that we proceed with the introduction of a terminology that affords a useful classification of the context embedding the constructs of interest.

## 3. Classification of Context: The Space-Time Continuum

As defined above, intelligence and creativity are both context-embedded phenomena, but the influence of context on them is distinctly diversified, so much so that it becomes the key allowing to understand when the two constructs overlap and when they can be distinguished. In order to develop a significant theoretical framework for the comparison of intelligence and creativity, it is therefore crucially important to introduce a clear and general taxonomy for the classification of the context embedding the episodes under study.

An “episode” is here defined as the conventional container for a specified human activity in a determined domain of knowledge, characterized by a thinking process and behavior that will be assessed in terms of its intelligence and/or creativity. As discussed in (Corazza 2019, 2020; Corazza and Lubart 2020), any episode is dynamically concatenated with past and future episodes, and as such the overall cultural evolution transcends the single individual to form a dynamic universal creative process.

### 3.1. Conceptual Space S and Available Time Span T

In line with (Corazza and Lubart 2020), we consider that there are two fundamental sociocultural dimensions that characterize any context embedding human episodes, respectively identified as the *conceptual space* S and the *available time span* T.

Given a specific episode in a certain domain of human activity, the conceptual space S should be intended as that portion of relevant knowledge that can be exploited and explored in the thinking process (Boden 2009; Newell and Simon 1972; Perkins 1992), in search for a tangible or intangible response. Domain specificity translates therefore into diversified conceptual spaces S, which should not however be considered as isolated silos, but rather as deeply interwoven epistemologic landscapes.

Any conceptual space S is clearly dynamic, expanding (or, more rarely, contracting, e.g., see Henrich 2004) as a consequence of individual and/or social intellectual activity in the relevant domain, through the fundamental human tools of ideation, communication, assimilation, followed by further ideation. As a consequence, any conceptual space S is intended to contain both extant knowledge in its domain and its possible expansions into what Kauffman (2016) identified as the *adjacent possible*. In other words, the outcome of the thinking process pertaining to an episode in conceptual space S is not necessarily a part of the extant knowledge, but may be characterized by originality (a necessary ingredient for a creative achievement).

On the other hand, the time span available for the episode, T, could be imposed by others or could be self-defined by the actor. Certainly, T is also subject to dynamic variations, in terms of expansion or contraction, in the course of an episode. In order to classify the fundamental characteristics of these two dimensions, we introduce here the attributes of *tightness* and *looseness*.

### 3.2. Applying Tightness and Looseness to Space S and Time T

Gelfand et al. (2011) introduced the concepts of tightness and looseness in order to classify the culture of nations. Briefly, a tight culture is characterized by very stringent norms, with little or no tolerance for those who break the rules. In a tight culture behavior is conventionally encoded and actively monitored, in the first place by institutions but also by peers. An exemplary tight culture is the one found in the Republic of Singapore (Gelfand et al. 2011). On the other hand, a loose culture is characterized by norms that are flexible and weakly applied, one where errors and violations are tolerated, so that people’s behavior is in general free and informal. A loose culture should not be intended as chaotic, but certainly less orderly, structured and controlled than a tight culture. A fitting example of a nation with a loose culture is given by New Zealand (Gelfand et al. 2011).

As discussed by Gelfand (2012), tightness and looseness are independent variables with respect to other cultural dimensions such as collectivism, individualism, power distance, or short- vs. long-term orientation; they turn out to be very useful in explaining several characteristics in a society, such as for example its innovation potential (Harrington and Gelfand 2014). In our theoretical framework, we borrow the concepts of tightness and looseness, however we do not apply them to general “culture”, but rather to the specific dimensions of conceptual space S and available time span T. This approach can be interpreted as a case of travelling concepts (Bal and Marx-MacDonald 2002) across disciplines.

Let us begin by considering a conceptual space S: our aim is to describe it as its characteristics vary from maximum tightness to maximum looseness. The “norms” in a specific conceptual space are given by the characteristics of the established knowledge in that domain, by the attitude of the gatekeepers, i.e., those experts who in various ways exert a form of control over the domain, as well by the situational and environmental elements. A tight conceptual space is therefore characterized by stringent norms in terms of what is conventional vs. eccentric, orthodox vs. outrageous, correct vs. incorrect: in the limit of tightness of a conceptual space S, to any problem or question there corresponds a single correct solution.

Therefore, a tight conceptual space is characterized by stringent constraints, and there is little or no tolerance for ambiguity or errors, especially by the gatekeepers but also by peers. This is not necessarily negative: when the tightness of a conceptual space is determined by environmental danger, a correct or incorrect response may be a question of life or death. In certain cases, the tightness of the conceptual space is such that it actively guides the actor to the solution: in the terminology introduced by Perkins (1992), this corresponds to a *homing space*, for which the structure of the problem indicates in itself the solution, allowing for the optimization of a response.

At the opposite extreme, a loose conceptual space S is characterized by the fact that it is possible to conceive many alternative responses, actions, solutions. In a loose conceptual space, there is little or no pre-conditioning of the outcomes of the episode, with ample possibility to accept shifts in paradigm, and high tolerance for ambiguity and errors. Referencing again Perkins (1992), a loose conceptual space is similar to the so-called *Klondike space*, an unstructured domain that is open for wide exploration, and where a-priori constraints are weak (see also Boden 2009).

Two examples are in order here to further clarify the tight/loose classification of a conceptual space S. Consider a mathematical problem. If the problem is posed correctly, and all the data are given, a single correct solution typically exists, and space S is tight. However, if the problem is ill-defined (e.g., underdetermined), and/or there are missing data, the mathematical space S becomes loose as multiple solutions (possibly infinite) exist. It should be noted that excessive formalism and logicism in mathematics, leading to extreme tightness in the discipline, has been criticized by Byers (2011) as a cause of depriving the discipline of its deeper meaning, to produce what Byers identifies as sterile certainty. As a second example, consider the goal of writing a poem. This is definitely a much looser domain than that of mathematics; yet, if the goal is that of writing a haiku, then the corresponding conceptual space S becomes relatively tight. In fact, a haiku is a three-line poetic form, originated in Japan, where the first line must have five syllables, the second line must have seven syllables, and the third line again must have five syllables. There exist uncountable possible haiku’s, but they all share this tightness of metric constraints. At the other extreme, free verse poetry does not impose either a consistent rhyme scheme, a metrical pattern, or a musical form, and therefore definitely corresponds to a much looser conceptual space for poem writing.

It would certainly be an interesting exercise to investigate the implications of tightness and looseness when the conceptual space S is enlarged to cover an entire disciplinary field. In this case, one way to interpret tightness vs. looseness could be in terms of the dominating schools of thought in a discipline, which may be tight if unified, insular, and firmly policed or loose if fragmented, porous, and contested. This kind of analysis was for example carried out in (Bender and Schorske 1998), where couples of disciplines where considered and compared over a period of fifty years (1945–1995) in the fields of humanities (Philosophy: tight vs. English: loose) and social sciences (Economics: tight vs. Political Science: loose).

Let us now consider the available time span T. A tight available time T means that the context imposes stringent requirements on the time span within which a response is expected to come out of the thinking process, and there is little or no tolerance for delay. In extreme conditions, even a correct response that is however affected by a delay can endanger one’s life, so much so that adhering to the time constraints can become a matter of survival. When time T is very tight, a delay in performance may be severely punished by the gatekeepers, by peers, or by institutions. In experimental conditions for psychological studies, time T is typically strictly controlled in order to ensure reproducibility and comparability of results. In professional endeavors, time T becomes tighter and tighter in view of incremental rates of activity by competitors.

At the opposite extreme, when the available time span is loose, the planning or scheduling of the episode is not strictly defined. Even if a deadline is hypothesized, there is ample tolerance for delays. In the case of absence of results, more time T can be made available for an episode, without direct negative consequences. When time T is loose, long periods of incubation for a problem are possible, such as those described by Henri Poincaré in his work on Science and Method (Corazza and Lubart 2019; Poincaré 1908), which also gave input to the famous four-stage model of the creative process (Wallas 1926).

### 3.3. Building the Space-Time Continuum

For any domain of knowledge and for both space S and time T, the characteristics of tightness and looseness will vary in a continuous way. Note that tightness and looseness in general have both objective connotations (e.g., a test with clearly identified problem space to be explored in a fixed amount of time) and subjective perception: in the same context, individual differences in personality and cognitive abilities may lead to differential perception of the tightness and looseness characteristics of space and time.

**Figure 2:**
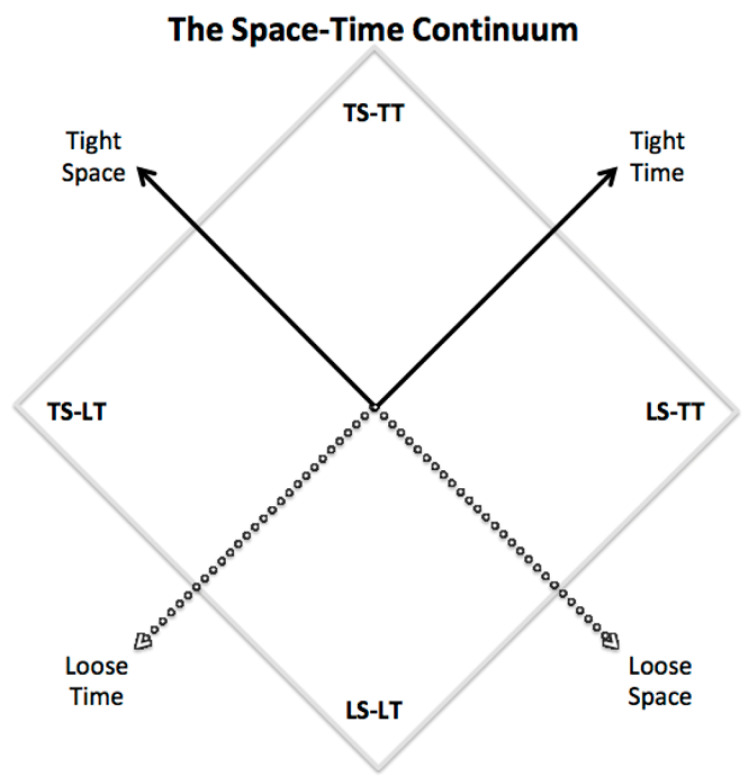
The four quadrants of the Space-Time Continuum.

Now, by considering these two dimensions as axes of a bi-dimensional space, we obtain what we identify as the *Space-Time Continuum*, pictorially represented in Figure 2. As shown, the space-time continuum can be subdivided into four quadrants:

- Tight Space-Tight Time (TS-TT) quadrant: pure tightness
- Loose Space-Tight Time (LS-TT) quadrant: hybrid looseness-tightness
- Tight Space-Loose Time (TS-LT) quadrant: hybrid tightness-looseness
- Loose Space-Loose Time (LS-LT) quadrant: pure looseness

As we will discuss in the following, these four quadrants correspond to distinctly different contexts that influence the actor’s behavior and our understanding of the corresponding intelligence and creativity constructs. As stated above, tightness and looseness for both conceptual space S and available time T vary in a continuous way, but in terms of discussing the prevailing trends in the four quadrants it is useful to move away from the origin and consider the main context characteristics as exemplified by the corresponding tightness/looseness of space S and time T, respectively. However, it should be noted that the midpoint break does not mean that there is any categorical difference on each side of the origin.

Before entering in the description of each quadrant, we note that context embeddedness produces a form of *pressure* on the episode under study, given by the combination of the two characteristics in terms of tightness/looseness of space and time. In general, tightness induces more pressure on the individual than looseness; also, tightness in time will generally produce more pressure than tightness in space. As a consequence, in the four quadrants pressure on the actor goes from maximum to minimum in the following order: (i) TS-TT; (ii) LS-TT; (iii) TS-LT; (iv) LS-LT. It is interesting to note that, in the classic 4P’s framework for the study of creativity introduced by Rhodes (1961), the effect of the environment is identified as Press; now we have introduced a taxonomy that allows to place the study of Press on both creativity and intelligence within a meaningful theoretical framework.

In the following, each quadrant of the space-time continuum is introduced with a quote by Albert Einstein (quotes were extracted on December 2020 from the website: wisdomquotes.com/albert-einstein-quotes/).

### 3.4. The Tight Space-Tight Time (TS-TT) Quadrant

“You have to learn the rules of the game. And then you have to play better than anyone else” (A. Einstein).

The space-time continuum picture in Figure 2 is purposely rotated by 45 degrees to show pictorially that the TS-TT quadrant is on top, i.e., metaphorically coherent with the fact that most of our lives (at least in Western and Far-East cultures) are populated by episodes that can be properly mapped onto this quadrant, in which the embedding context is tight in both conceptual space S and available time T. This applies to most educational, academic, and professional environments.

In this kind of context, the conceptual constraints are strong and the time pressure is high, so that the actor is induced to search for the best solution to any problem he/she might face in the minimum possible amount of time. There is little tolerance for conceptual ambiguity or delay. This quadrant is the home of correct and fast responses.

Let’s consider the intelligence and creativity constructs in this context, according to their definitions DI and DC reported in Figure 1. When conceptual space S is tight, it is extremely important to have clearly defined goals, that must be pursued, and hopefully achieved, as efficiently as possible in the minimum amount of time. Therefore, an intelligent behavior as defined in DI is perfectly adaptive and necessary in the conditions provided by the TS-TT quadrant.

In fact, traditional school and academic programs are designed to bring everyone’s intelligence to a sufficient level in order to lead an intelligent life in tight space-tight time contexts. Measuring IQ (e.g., Caemmerer et al. 2020; Kaufman 2009) produces conditions for the participants that are tight in both S and T, which are perfectly suited to elicit the abilities and attitudes related to the intelligence construct.

In contrast, considering creativity as defined in DC, it is evident that in TS-TT conditions the potential for originality is in general very low. There are both epistemological obstacles, given by the tightness of the conceptual space S, and emotional obstacles, provided by the punishment associated with failing to provide a correct answer in due time. In general, creative behavior in the TS-TT quadrant is maladaptive, and individuals showing this kind of behavior may be judged as distracted, slow, ineffective, or in the limit as rebels refusing to follow the rules.

### 3.5. The Loose Space-Tight Time (LS-TT) Quadrant

“Creativity is intelligence having fun” (A. Einstein).

Considering the LS-TT quadrant in Figure 2, the available time span is tight but the constraints and expectations on the conceptual space are loose, with ample possibilities for exploration of alternatives. Quadrant LS-TT is hybrid in its characteristics. It is a type of context characterized by a sense of urgency: time pressure is high, and there is little or no tolerance for delays; however, the problem the actor is facing is open-ended and allows a multitude of possible responses. Pressure on the actor is very high, in certain cases even larger than in quadrant TS-TT, because results are wanted within a strict deadline but for a task with multiple possible responses.

Considering a professional environment, the LS-TT quadrant represents well the typical context in which product and service innovation has to be pursued: time-to-market is at a prime, the competition must be anticipated, but the future evolutions of user needs are uncertain, opening up a multitude of alternative routes that can only be evaluated a-posteriori. A mistake could have very severe consequences on a business, which contributes to the very high pressure in this quadrant. This quadrant can be considered to be the home of short-term innovation. It should be noted that the presence of time constraints is not necessarily detrimental to creativity, as discussed in Beghetto (2019).

From the point of view of the dynamic definition of creativity DC, these are the typical embedding conditions for a process of time-pressed exploration of alternatives which should have both a potential for originality and a potential for effectiveness. Creative achievements will be determined a posteriori, typically in the near future, and success will not only depend on the intrinsic qualities of the conceived ideas, but also on the activity of competing actors and on the variable trends in the final consumers and customers. A creative behavior, as defined by the dynamic definition DC, is adaptive in this quadrant.

On the other hand, considering the definition of intelligence DI, even though the specific solutions are not-predetermined in a loose space S, there is still the possibility to set as a goal the provision of as many alternatives as possible in a stringent time frame. The efficient pursuit of this goal would therefore correspond to an intelligent behavior, leading to a significant overlap of the creativity and intelligence constructs in this quadrant. In other words, high performance embedded in a LS-TT context requires high levels of both intelligence and creativity. Given the importance of this quadrant for the professional domain, this is a clear indication of the necessity for the development of both constructs at all levels of education, including continuous life-long education.

### 3.6. The Tight Space-Loose Time (TS-LT) Quadrant

“It’s not that I’m so smart, it’s just that I stay with problems longer” (A. Einstein).

Moving to the TS-LT quadrant in Figure 2, the constraints and expectations in the available time span T are released, whereas conceptual space S is tight. Quadrant TS-LT is also hybrid in its requirements, as is quadrant LS-TT, but with dual characteristics. The tasks and problems facing the actor are highly structured and range typically from high to very high complexity, so that they cannot be solved in a short time, and it may be hardly possible to impose pre-determined deadlines. Hence, space is tight but time is loose.

As an example, trying to tackle a mathematical problem that has remained unsolved for many years, decades, or even centuries, would elicit a TS-LT episode. The value of finding effective solutions in these contexts is in general very high. The TS-LT quadrant may be considered to be the home of complex problem-solving. It also corresponds to all contexts in which human activity is not expected to produce immediate benefits in the short-term, but is rather pointing to results in the medium- or long-term. In the TS-LT quadrant there is a form of investment in the longer term that allows a reduction of the pressure for immediate results, freeing up the possibility to stay with problems longer.

In terms of intelligence, the conceptual space S being tight, the goals can still be defined precisely, and their efficient pursuit and achievement will correspond to the individual gaining a very strong reputation for being intelligent, or in the limit a genius. In terms of creativity, in these conditions the solution for the complex problem is not known a-priori, so its search and conception will always be characterized by a very high potential for originality.

In essence, in this quadrant the intelligence and creativity constructs largely overlap, although intelligence might have the prevalent role, creativity being often confined to the eureka moment of intuition. Perhaps one of the most fitting examples in this quadrant is the aforementioned thinking style of Henri Poincaré (Corazza and Lubart 2019).

### 3.7. The Loose Space-Loose Time (LS-LT) Quadrant

“Everything that is really inspiring is created by the individual who can labor in freedom” (A. Einstein).

Finally, quadrant LS-LT is a mirror-image of the first quadrant we examined, TS-TT, representing a context whereby both conceptual space S and available time span T are loosened. This quadrant is the home of free and unscheduled exploration, a context that can be considered to be fortunate and rare in modern societies. This is the quadrant of the space-time continuum in which pressure on the actor and on the episode reaches its lowest values. The danger of such a low level of a-priori expectation and pressure takes the form of possible wastes of time and resources.

Considering the dynamic definition of creativity DC in Figure 1, this is the context in which tolerance for creative inconclusiveness is maximum. Therefore, in this quadrant the distinction between the dynamic definition of creativity and the standard definition is both clear and fundamental. The actor is allowed to try, modify, refine, fail many times, and the level of punishment is in general low, and certainly much lower than in any of the other quadrants. Creative achievement is certainly welcome, but its pursuit is not dictated by stringent time schedules (nor could it be).

As well represented by the Einstein’s quote reported above, this is the context that allows (but certainly does not guarantee) the highest forms of creativity, possibly leading to paradigm shifts and opening up seminal new areas of human thought. In this quadrant there is typically no evident problem to be solved. If there were, this would immediately translate into a form of pressure if the problem were an important one, transferring the activity into one of the other quadrants, depending on the nature of the problem itself. In some cases, the outcomes of the activity in the LS-LT quadrant can be hardly appreciated by the outside world, and recognition could only come posthumously.

Consider now the definition of intelligence DI and its implications in this quadrant. In this context, there is ambiguity in the definition of the goals. The lack of constraints allows a multitude of alternatives in the objectives that the actor might wish to pursue, and much is left to the responsibility of the individual. Even if the goals are defined and efficiently pursued and achieved, the outside world might not notice or give any form of praise or recognition. Satisfaction might be deferred indefinitely, and this can produce various forms of unhappiness in the actor who is not ready or willing to live for long times in a state of inconclusiveness.

Given this potential impact on well-being and happiness, it may be deemed wiser and more intelligent to move one’s activity in another context, one in which pressure is higher because the goals are shared by a large and important part of society. In essence, in the LS-LT quadrant a creative behavior is adaptive, whereas an intelligent behavior is likely to produce a move to another quadrant.

### 3.8. Intelligence and Creativity in the Space-Time Continuum: Basic Mapping

The above discussion is intended to show that the use of the dimensions of conceptual space S and available time span T, as they respectively vary from tightness to looseness, affords the introduction of a very clear taxonomy and possibility for a classification of the embedding context that provides clear directions for understanding the relationship between the intelligence and creativity constructs.

**Figure 3:**
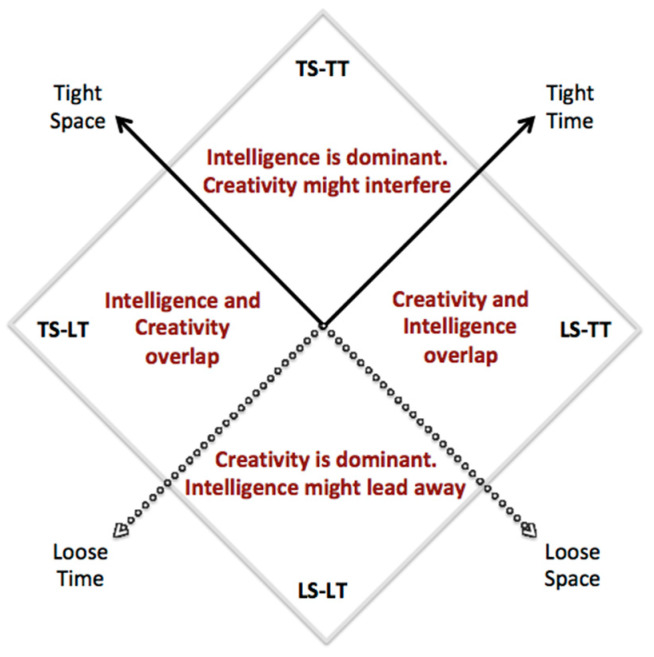
Basic mapping of intelligence and creativity construct over the space-time continuum.

As represented in Figure 3, a basic mapping of the intelligence and creativity constructs can be produced. As it represents a simplification, this should be taken with caution for the following two reasons. First, tightness and looseness vary continuously, so that this basic classification should be intended as the limiting condition corresponding to a discretization of scales.

Second, as anticipated before, the perception and the extent of the tightness and looseness of space and time is subject to individual differences, as well as to societal differences. As a simple example, a specific problem might be felt to be very complex or very simple depending on the expertise of the actor in the relevant domain. A second example can be given by those situations in which an individual has the power to (at least partially) moderate the tightness and looseness of its environment, such as the chief executive officer of a company.

Having clarified these precautions, with the aid of Figure 3 we can state the following general indications:

- In the TS-TT quadrant the intelligence construct is dominant. The pursuit of creative behavior might interfere in the success of an individual, leading to distraction and/or delay, which can result in forms of punishment. This context represents the majority of situations encountered in the modern life of individuals, at least in Western and Far-East developed cultures. For this reason, education systems in these cultures have been typically designed for this kind of context and for the development of intelligence, although a need for creativity is clearly growing (Corazza 2017).
- In the LS-LT quadrant the creativity construct is dominant. It is essential to introduce the dynamic definition of creativity in order to have a complete theoretical framework, for in this quadrant long periods of creative inconclusiveness may be experienced (these would be missed under the SD static creativity framework). Given the inherent harshness of such situations, and the suffering often associated with them, a value placed on intelligence might lead away the actor, in the sense that it might push the individual to change his/her environment, searching for alternative contexts which are more readily adaptive for one’s wellness.
- In the two hybrid LS-TT and TS-LT quadrants the intelligence and creativity constructs largely overlap, and it may be very difficult to discriminate them from each other. A slight prevalence of intelligence can be argued for in the TS-LT quadrant, given the tightness of the conceptual space S, whereas a slight preference for creativity can be argued for in the LS-TT quadrant, given the emphasis on the search for alternatives. At any rate, the cognitive and emotional components underlying intelligent and creative behavior in these contexts should show significant levels of interaction and overlap.
- For the purpose of distinguishing as clearly as possible between the two constructs, it is therefore useful to concentrate on the “pure” TS-TT and LS-LT quadrants. For the purpose of finding commonalities between the two constructs, it is useful to concentrate on the “hybrid” LS-TT and TS-LT quadrants.

In the above discussion, the context is intended as the actual environment within which an episode occurs, where the intelligence and creativity constructs may manifest themselves. For the purposes of observation, it may be useful also to create controlled episodes in laboratory environments. We can therefore introduce a distinction between *actual-contexts* and *measurement-contexts*. The latter are discussed in the following section.

## 4. Measuring Intelligence and Creativity in the ST-Quadrants

Having discussed the importance of context over the manifestations of the intelligence and creativity constructs, it should be clear that a scientific understanding of their mutual relationship must be based on a scientific measurement approach which also takes context into account.

It is important to underline that any measurement, irrespective of its form, presupposes and induces a context within which the individual is immersed: we identify this as the *measurement-context*. When observations are made in the field, the measurement-context corresponds directly and almost completely to the actual-context embedding the episode, with the only possible exception of the interference introduced by the observer (a form of Heisenberg’s uncertainty principle as applied to human studies).

On the other hand, other conditions, such as for example experimental tasks in a laboratory, create a very specific measurement-context (which is nonetheless a real context embedding the episode) that might correspond only indirectly to other actual contexts. In these cases, it is fundamental to establish the desired predictive validity of the measurement, i.e., the possibility to take the results obtained in the measurement-context and extrapolate their validity to the actual-context under study.

In general, the level of consonance between the characteristics of the actual and measurement contexts will be an important element in determining predictive validity. For the purpose of allowing reflection on this level of consonance, it is possible to apply the classification based on the notion of the space-time continuum also to the measurement-context.

### 4.1. Measuring Intelligence and Creativity in a TS-TT Context

It can be immediately stated that a tight-space and tight-time measurement-context is ideally suited for laboratory experiments and tests. First of all, a tight control of T is necessary to ensure that all participants are engaged for the same available time. If this were not the case, it would become very difficult if not impossible to compare results and include them in a single statistical analysis. On the other hand, the instructions given to participants contribute to making the conceptual space S tight, by restricting the allowed paths for thought to enhance focus and precision of measurement. The more the set of possible responses is structured, the tighter the conceptual space S becomes, reaching a maximum when a single correct response exists and punishment follows incorrect answers.

Examples of TS-TT contexts for measuring intelligence are provided in the vast majority of intelligence tests. The most prominent example is undoubtedly given by IQ testing (e.g., Kaufman 2009; Wechsler 1949, 2008), followed in importance by SAT (Zwick 2013, and references therein), the impact of which on college admission has been the subject of intense debate for many years (see for example Grissmer 2000). Many other more specific intelligence tests exist, among which Raven Progressive matrices (Raven 2000; Raven et al. 1998), the Intelligence Structure Battery (Arendasy et al. 2004), the Kit of Factor-Referenced Cognitive Tests (Ekstrom et al. 1976); the list could definitely go on much longer.

The neuroscience of intelligence is an extremely important field of investigation, searching for neural correlates of intelligent behavior (see Haier 2016, for an extensive overview). The difficulty of observing the brain’s behavior calls for further tightening of both space S and time T, so that these experiences properly belong to the TS-TT quadrant.

In contrast, examples of TS-TT contexts for directly measuring creativity are quite difficult to identify. This is because the tightness of this context greatly reduces the potential for originality and effectiveness of any episode: there is very little freedom for exploring, and it might be detrimental to performance to do so. At any rate, because convergent thinking is considered to be an important part of the creative thinking process, this is the kind of component that can be measured in the TS-TT context.

An often-cited test for the convergent thinking component of the creative process is the Remote Associates Test, or RAT (Mednick 1968). In a RAT episode, the participant is asked to find in a predetermined time frame *the* word that is semantically associated with a given set of words (typically three to five). Note in any case that, in line with our observation that the TS-TT quadrant is largely dominated by intelligence, the question about whether RAT measures creativity or intelligence has been raised (Lee et al. 2014). The distinction might lie in the cognitive strategy adopted by the individual in solving the task, involving either an analytical search or a sudden insight.

### 4.2. Measuring Intelligence and Creativity in a LS-TT Context

In this quadrant, time T is tightly controlled as in the previous section; hence, it is still a condition that is readily amenable for laboratory experiments and tests. However, given that the conceptual space S is loose, the concerned measures must allow the participant to explore a variety of alternatives.

Given that time is still tight, the pure number of alternatives that the participant is able to explore is already an important figure of merit in this quadrant, identified as *fluency*. Several tests for intelligence measure fluency, see for example the already cited Kit of Factor-Referenced Cognitive Tests (Ekstrom et al. 1976). To focus on a specific ability, in this kit word fluency is defined as “*The facility to produce words that fit one or more structural, phonetic, or orthographic restrictions that are not relevant to the meaning of the words*” (Ekstrom et al. 1976, p. 73). The task may then be to write as many words as possible beginning, or ending, with certain given letters. Clearly, no *originality* is expected as an outcome of this item, hence it cannot be considered to be measuring creativity, but only a form of retrieval from memory.

Viceversa, whenever the test includes (explicitly or implicitly) a request for originality, then we move into the field of measuring the creativity construct, in its divergent thinking component (Runco and Acar 2012). A classic test for divergent thinking, which fits perfectly into the LS-TT quadrant, is the Unusual Uses Test (UUT) or Alternative Uses Test (AUT), first introduced in (Christensen et al. 1960) and then inside a complete test battery by Guilford (1967), designed to be in line with his structure-of-intellect model (Guilford 1956, 1988). It is worth noting that when originality is explicitly requested by the AUT instructions, the available time T must be longer than that foreseen without an originality requirement: tightness in T must be calibrated on the task goals.

A third dimension that can be measured through AUT is *flexibility*: the capacity to switch from semantic category to semantic category during ideation (Acar and Runco 2017; Finke et al. 1992). This is an important ability that can only be measured if the context is loose.

The neuroscience of creativity is an emerging field of investigation searching for the functional significance of the neural correlates of creative behavior (Abraham 2018; Benedek et al. 2019; Jung and Vartanian 2018). The requirements of investigation for the neuroscience of creativity are very similar to those for the neuroscience of intelligence: time T must be strictly controlled, the task must be specified very clearly, although in this case the conceptual space S must be loosened enough to allow a potential for originality to exist. Typically, the AUT test is adopted, and it is possible to find statistically significant connections between brain waves power in specific bands and brain structures and the originality scores provided by external judges (Agnoli et al. 2020). The neuroscience of creativity is a field of investigation that maps well inside the LS-TT quadrant. The pursuit of observing and understanding the neural correlates of both constructs (Benedek et al. 2018) spans across both the TS-TT and LS-TT quadrants.

Now, a crucial point needs to be discussed whenever one addresses measurement of the creativity construct in a loose space S: given the definition of creativity DC reported in Figure 1, the potential for originality of a process outcome may be evidenced through a form of appreciation by the outside world, but this would be subject to dynamics in time, space, culture. This apparent lack of objectivity in the measurement of originality is clearly a problem in testing, especially when the objective is to build statistics over large samples.

Wallach and Kogan (1965) tried to find a solution to this difficulty by intending originality as *uniqueness* or *relative infrequency* of the response inside the set of alternatives produced during the experiment. This very simple way of scoring originality had a large impact over the research community, but is unfortunately dramatically biased: in fact, the uniqueness of a response correlates positively with fluency (the larger the number of responses, the higher the probability that at least one is unique) and negatively with the sample size (the larger the sample size, the smaller the probability that any response remains unique). As observed in (Silvia 2015), response uniqueness is confounded by both fluency and sample size.

Therefore, the creativity research community has introduced subjective ratings by external judges, typically two or three. In this case, the difficulty might come when judges do not agree: for this reason, the Consensual Assessment Technique (CAT) has been introduced (Amabile 1982) and used extensively (Kaufman et al. 2008). It is normal practice to train judges in order to reach a desired level of inter-rater agreement.

Whereas this is a very sensible protocol for testing in a LS-TT measurement context, we can note that in the actual field the creative process happens in a different way: the higher the potential originality of an outcome, the larger the disagreement even between the experts in the relevant domain. This is the reason why the notion of disruptive innovation has been introduced. A paradigmatic example would be *Ulysses* by James Joyce (1922), where, as Jung (1953) noted, the traditional criteria for beauty and meaning, held as valid at the time of writing, were destroyed. Clearly, *Ulysses* caused scandal, outrage, and astonishment in the critics, provoking a segmentation in two fronts: those who discarded the book in disgust, and those who praised the paradigm shift it had brought about. This radical shaking of the field of pertinence can be considered as the signature of those creative outcomes that lead to paradigm shifts.

In closing this section, we should mention the fact that measuring divergent thinking is only one possibility for testing components of the creativity construct in the LS-TT quadrant, but others exist (Barbot et al. 2019). As an example, another important measure of integrative synthesis is given by the test for creative thinking-drawing production (TCT-DP; Urban 2004). Participants are given a set of graphical elements and a fixed amount of time (e.g., 15 min) to draw a picture that uses all the elements. In scoring the result, unconventionality is at a prime, which depends strongly on giving the elements a new meaning with respect to their usual signification.

In a related line of work, Lubart et al. (2011) developed the EPoC battery, to Evaluate Potential Creativity in children and adolescents. This test consists of both divergent-exploratory and convergent-integrative tasks in a range of content domains (graphic, verbal-literary, social, mathematics, musical, scientific, and body movement). Each task presents a selected set of stimuli that the respondent must use in a limited time. The comparisons allow a child to be scored with respect to the reference population (in the same country), with fluency for divergent-exploratory tasks and judges’ ratings for convergent-integrative tasks. For example, in the verbal-literary domain, a divergent task requires multiple endings of a story to be proposed whereas a convergent task involves generating an original story based on three characters provided.

### 4.3. Measuring Intelligence and Creativity in a TS-LT Context

In the tight-space and loose-time quadrant the person’s mind moves in a highly structured conceptual space S, which in terms of measurement affords to achieve a level of objectivity in scoring the outcomes of the episode under study that is not possible to obtain when space is loose. However, the available time span T is loose: this means that the aforementioned outcomes can be generated at time instants that are not tightly scheduled.

Loosening the time dimension renders it therefore very difficult to perform observations in the laboratory, and shifts the efforts of scientific research towards the field. In these conditions, the methodology for investigation must change, giving emphasis to approaches that are able to capture trajectories over longer periods of time, such as case studies (Ericsson 2014; Ruthsatz and Detterman 2003; King et al. 2019; Wallace and Gruber 1989), biographies of persons showing eminence in tight conceptual spaces (Fölsing 1997; Pickover 2008), diary studies (Bolger et al. 2003; Mehl and Conner 2012), experience sampling methods (Hormuth 1986; Larson and Csikszentmihalyi 2014), historiometric studies (Simonton 2006, 2009, 2016), dynamic testing (Feuerstein 1979; Sternberg and Grigorenko 2002; Vygotsky 1980), and other methods that involve unstructured time spans.

It should be noted that longitudinal studies are not necessarily classified under the TS-LT quadrant: as an example, suppose that an IQ test is administered to a population sample twice at a distance of ten years; in this case, what has happened is that two instances of TS-TT measurement have occurred, allowing the researcher to observe longitudinally the evolution of tight-space and tight-time performance of the sample. On the contrary, TS-LT measurement approaches should allow the person under observation to be engaged in episodes that are characterized by a loose available time span.

Finally, it should be evident that pure measurement of a single construct in these conditions is in general very difficult, because many interactions occur in a loose T episode, so that personality characteristics and social elements become relevant in determining the outcome. However, this is a price that must be paid when we want to observe episodes in the real world, in the field, for extended periods of time.

### 4.4. Measuring Intelligence and Creativity in a LS-LT Context

Measurement reaches its highest level of difficulty when both space S and time T become loose. All the considerations about the necessity to make observations over time periods that allow the dynamics of an episode to take place, as discussed under the TS-LT quadrant, apply here as well. However, in addition, the difficulty in assessing the outcomes of the episode in terms of its potential originality, that we discussed for the LS-TT quadrant, now must be taken into consideration.

Undoubtedly, measuring reliably intelligence and creativity in the LS-LT quadrant is the most difficult and ambitious objective. For the same reasons, it is a very interesting problem area, in which there appears to be room for the definition of innovative measurement procedures. The measurement approaches that have been listed under the TS-LT quadrant can all be considered here, but they have to be re-considered in terms of the difficulties associated to capturing the important elements of behavior in a loose conceptual space.

For example, consider the study of eminent creative persons in Lebuda (2014): even the selection of the persons to be considered in the study is affected by serious difficulties, in terms for example of cross-cultural variations and of confusing popularity for eminence.

These nuances in judging creativity constitute one of the main reasons for introducing the dynamic definition of creativity, of which definition DC in Figure 1 is an evolution. Accepting as a given the additional level of difficulty associated with measurement in the LS-LT quadrant, examples of measurement and observation approaches can be found for biographies of eminent persons working in loose conceptual spaces (Galenson 2004; Lebuda and Csikszentmihalyi 2020; Simonton 1978), diary studies (Botella and Lubart 2019; Botella et al. 2019; Karwowski et al. 2017), self-reporting of achievements over extended periods of time (Carson et al. 2005; Wallach and Wing 1969), historiometric studies of creativity (O’Connor et al. 1995; Simonton 1999), case studies (Hanson and Glăveanu 2020; Wallace and Gruber 1989).

## 5. Discussion and Conclusions

Once the definitions for intelligence and creativity are declared and their mapping on the space-time continuum described, along with possible quadrant-specific measurement methodologies, the proposed theoretical framework is ready for exploitation. It is clearly not possible to address all its consequences here, so only an initial discussion for possible areas of investigation is proposed.

### 5.1. Theories and Hypotheses on Intelligence and Creativity

The first point to be addressed is the relationship between the space-time continuum and existing theories and hypothesis on intelligence and creativity, as well as their relationship.

The Cattell–Horn–Carroll (CHC) model is arguably the most accepted one for a factorial representation of the abilities related to intelligence (Carroll 1993). A few CHC factors have already been studied in association with creative performance, such as fluid intelligence (Gf), crystallized intelligence (Gc), long-term retrieval (Glr), and processing speed (Gs). Now the open question is to understand how these associations are affected by the context as described by the space-time quadrants.

For example, we can hypothesize that speed processing is likely to be fundamental whenever time T is tight, but less so when T is loose. Further, there may be prevailing importance of Gc when space S is tight, but crystallized intelligence will be necessary in all quadrants because a minimum amount of knowledge is required for any cognitive process to operate upon.

However, based on the definitions in Figure 1, it should be noted that intelligence is a function of knowledge to produce correct evidence, whereas creativity is a function of knowledge to produce potentially original evidence. As a consequence, it is reasonable to expect that a threshold hypothesis TH should hold, but it would not be reasonable to expect that a single IQ threshold should exist for all testing conditions in all quadrants (Karwowski and Gralewski 2013; Jung et al. 2009).

Furthermore, prevailing relevance of Gf should increasingly emerge when space S becomes looser and looser. Based on these observations, we can hypothesize that a TH is more likely to be expected based on Gc than on Gf, which is indeed in line with results by Sligh et al. (2005). The relationship between Gf and divergent thinking performance in the LS-TT quadrant was analyzed in (Miroshnik and Shcherbakova 2019), but revealed only weak levels of correlation.

The necessary condition hypothesis NCH, according to which intelligence is a necessary (but not sufficient) condition for creative achievement is also certainly recognizable in the four space-time quadrants, but with variable significance depending on the characteristics of space and time. E.g., we could hypothesize that intelligence is necessary *and* sufficient in the TS-TT quadrant, and then intelligence should remain a necessity but its degree of insufficiency should grow as we move respectively into the TS-LT, LS-TT, and LS-LT quadrants. In particular, in the loose-space loose-time quadrant, when intelligence grows beyond the minimum necessary, it might drive the actor away from the effort: this observation, if confirmed, would be in line with the so-called interference hypothesis: at high levels of creativity, intelligence may be interfering (Sternberg and O’Hara 1999).

We mentioned already the model of multiple intelligences by Gardner (1987): this is definitely compatible with the proposed theoretical framework based on the space-time continuum, considering that each dimension proposed by Gardner would correspond to a different space S, and that the tightness-looseness characteristics are themselves a function of the specific domain (e.g., compare linguistic vs. logical-mathematical). The detailed definition of this dependence is a path for future work.

Of course, also in the case of creativity it is very important to address domain specificity, i.e., the dependence of attitude and performance upon different conceptual spaces S for creativity episodes (for a review across many domains see Kaufman et al. 2017).

Mapping the triarchic theory of successful intelligence by Sternberg (2018) and its augmented version (Sternberg 2020) on the space-time continuum is also a very important path for research. It can be hypothesized that the importance of the analytical component will be dominant in the TS-TT quadrant, and remain important in all other quadrants in a similar fashion as the NCH discussed above. The practical and creative components, that allow the actor to select and shape his/her environment, refer to the fundamental interaction between the context and the individual, and this is definitely an area requiring further investigation.

Further theories that should be discussed in their relation to the space-time continuum include, among others, VPR (Verbal, Perceptual, and image Rotation) by Johnson and Bouchard (2005), PASS (Planning, Attention, Simultaneous and Successive processing) by Naglieri and Das (1990), PPIK (Process, Personality, Interests, and Knowledge) by Ackerman (1996), the Amusement Park model for creativity by Kaufman and Baer (2004).

Finally, we should mention the fundamental importance of metacognition for both intelligence and creativity (Kenett et al. 2018). However, the role of metacognition should be different for the two constructs, in line with their different definitions provided in Figure 1. The hypothesis could be that whereas for intelligence metacognition is fundamental to inhibit instinct responses, in a pursuit of rationality, in the case of creativity metacognition serves to inhibit conventional responses. Clearly, the importance and characteristics of the above functions should change as a function of the tightness vs. looseness of both space S and time T, and it would be important to investigate how.

### 5.2. Eminent Personalities and Personality-Related Variables in the Space-Time Continuum

Eminent persons who excelled in intelligence and creativity can be mapped onto the quadrants of the space-time continuum, in order to further exemplify significant typologies of contribution. As examples: Pico della Mirandola and Marilyn Vos Savant for the TS-TT quadrant, in view of their excellence in terms of, respectively, memory and IQ; Guglielmo Marconi and Steve Jobs for the LS-TT quadrant, in view of their extraordinary capacities of bringing to life disruptive innovations working on a tight schedule, to beat competition; Marie Curie and Henri Poincaré for the TS-LT in view of their undisputed career success as scientist and mathematician, able to tackle problems of the highest complexity; finally, Leonardo da Vinci and Vincent Van Gogh as heralds for the LS-LT quadrant, as they were both able to produce unprecedented creative work while living in unfortunate and under-appreciated conditions, apparently driven by a longing for future recognition.

These studies of eminent cases would then lead the way to a more general investigation of personality in the space-time continuum, to identify those traits that render an individual behavior more or less adaptive to the context. For example, adopting the Big-5 terminology (McCrae and Costa 2003), it can be hypothesized that high conscientiousness is very adaptive in the TS-TT quadrant, but also in the TS-LT quadrant, in combination with persistence. In contrast, openness to experience (Corazza and Agnoli 2020) can be hypothesized to be at a prime in the LS-TT quadrant, but even more so in the LS-LT quadrant.

Further, the influence of tolerance of ambiguity and need for closure will certainly have a role in determining whether an individual feels comfortable in a certain context. Loosening space requires tolerance of ambiguity. Tightening the available time span induces a strong need for closure. Additionally, it is clear that emotional intelligence (Petrides 2011) will play a key role in the management of the variable levels of pressure and frustration that can be experienced in the various contexts (Agnoli et al. 2019; Petrides et al. 2004).

Finally, self-concept has been shown to be key in complex problem solving beyond reasoning abilities (Christ et al. 2020). All of these elements will interact with the intelligence and creativity constructs to deliver an indefinite range of individual differences, which form an area of research of extreme interest.

### 5.3. Development, Education, and Professional Contexts in the Space-Time Continuum

The context in which an individual is brought up has fundamental consequences on his/her development. It can be hypothesized that the dominant characteristics of the space-time continuum in which an individual actually lives may be *internalized* to become a thinking style, thus favoring the flourishing of intelligence, of creativity, or both.

This process of internalization is in line with that proposed by Vygotsky (1980), according to whom interpersonal processes experienced in the environment (society, organization, family, school) can gradually become intrapersonal, and then act to develop intelligence and creativity, as well as determine behavior, goal setting, decisions, life plans. This would represent the qualitative feature of human intelligence.

However, the above should not be intended as situationism (Skinner 1955–1956), but rather as interactionism (Bowers 1973). In fact, the situationist approach would tend to discredit the person as an autonomous active agent. Viceversa, according to Bowers (1973, p. 327): “*Situations are as much a function of the person as the person’s behavior is a function of the situation*.” It is impossible to completely separate the context from the person observing it, or living in it. In Piagetian terms, it is not only a process of assimilation of a context but also of accommodation of the context itself. In perceiving or actively modifying the context, one expresses his/her individual differences. Moreover, there is a considerable consistency in the kinds of contexts that people create for themselves. One tends to “prefer” a certain kind of context, which will reinforce a specific type of behavior influenced by that specific context.

In essence, there is at the same time a context-specific character of behavior and a person-specific character of context. Interactionism considers that “context” is constructed, emerging out of a dynamic balance between assimilation and accommodation, between person and environment.

Designing the context for educational experiences in terms of the tightness-looseness of the involved conceptual space S and available time T can therefore be hypothesized to bear critical consequences on the development of both intelligence and creativity of pupils. Furthermore, the consonance or contrast between the context one experiences in the family and at school will largely determine success in educational endeavors.

As an example, Heath (1983) conducted an ethnographic study of language development in American schools, considering pupils brought up in different towns and communities (respectively Roadville and Trackton). Evidence emerged that those children raised in the Roadville environment showing a rigid structure in both time and space performed well in school, as opposed to Trackton children who lived considerable looseness in their family environment, and generally performed quite poorly at school. Clearly this avenue for research is quite ample and promising.

Finally, considering professional environments, it would again be possible to adopt the taxonomy of the space-time continuum to model and analyze the climate and culture that pervades work in a company, to extract information about how much and in what way intelligence and creativity are valued in that specific context. This could be related to the performance of the company in terms of production of intellectual property, but it could also be very useful in terms of recruiting personnel, creating testing and interviews approaches with specified space-time characteristics, oriented to the measurement of the intelligence and creativity constructs of the candidates.

In conclusion, we believe that the presented theoretical framework based on the proposed definitions for intelligence and creativity and their mapping on the space-time continuum serves its purpose of contributing to clarifying the relationship between the two constructs, indicating conditions for mutual overlap or distinction, and opens the way to many possible avenues for scientific research.

## Data Availability

Not applicable.
